# Sarcolipin Makes Heat, but Is It Adaptive Thermogenesis?

**DOI:** 10.3389/fphys.2018.00714

**Published:** 2018-06-14

**Authors:** Kevin L. Campbell, Alysha A. Dicke

**Affiliations:** ^1^Department of Biological Sciences, University of Manitoba, Winnipeg, MB, Canada; ^2^Technology Specialist, Fish and Richardson P.C., Minneapolis, MN, United States

**Keywords:** sarcolipin, thermogenesis, evolution, muscle, calcium, SERCA

A regulatory protein (sarcolipin or SLN) bound to SERCA pumps in the sarcoplasmic reticulum (SR) of cardiac and skeletal muscle has been shown to increase heat production *in vitro*. Here, we review recent work in this area, assess the potential of *in vivo* heat generation by this mechanism, and advocate that a comparative approach is the best path toward resolving many outstanding questions on the physiological function, regulation, and evolution of SLN.

## Introduction

**Figure 1:**
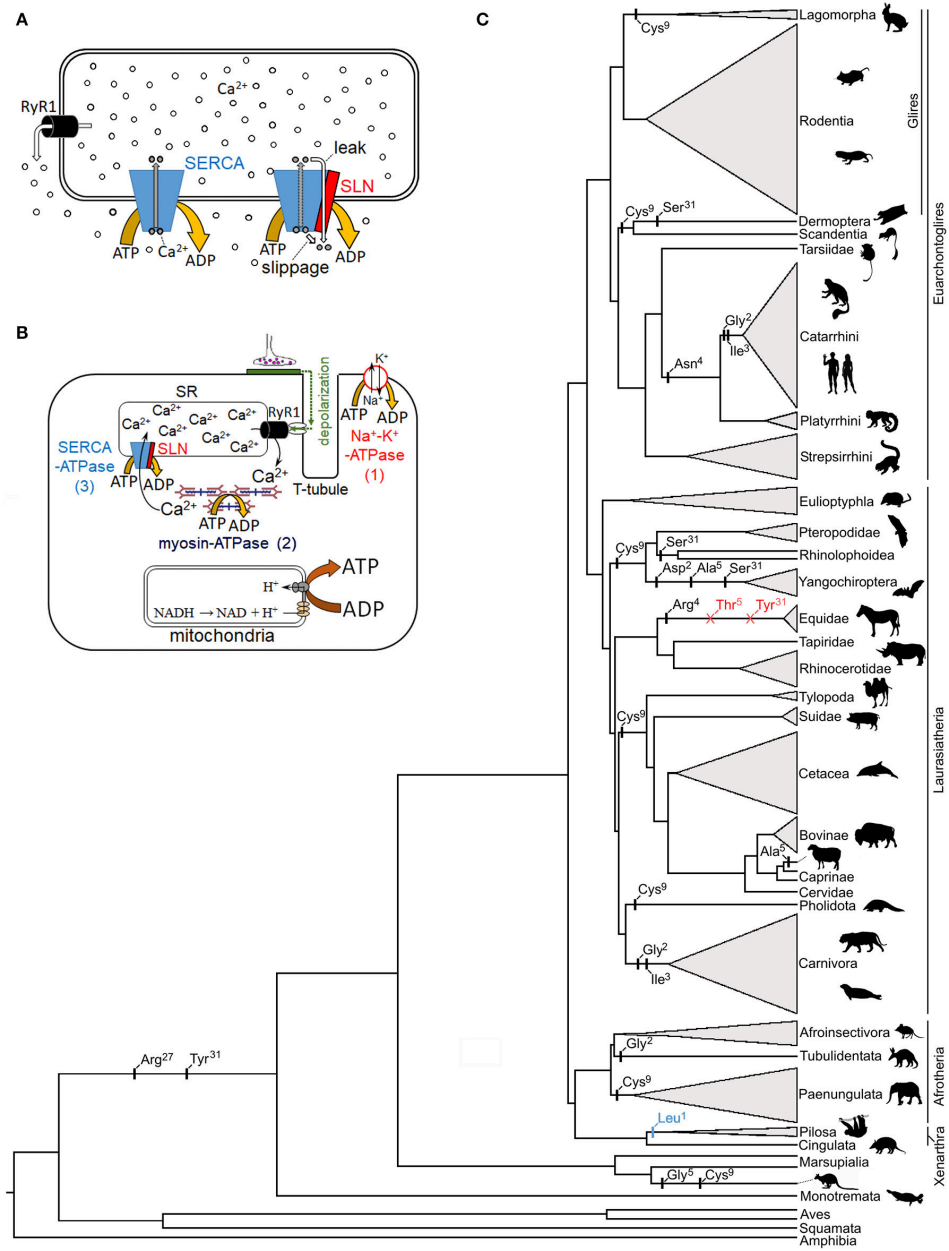
Proposed mechanism of sarcolipin in skeletal muscle non-shivering thermogenesis, and its genetic evolution**. (A)** The two functional mechanisms by which SLN is postulated to return SERCA-bound Ca^2+^ to the cytosol following ATP hydrolysis: enzyme slippage and passive leak (*right complex*). **(B)** Primary mechanisms of ATP hydrolysis in a typical skeletal muscle fiber following a nervous stimulation: (1) Na^+^-K^+^-ATPase, (2) myosin-ATPase, and (3) SERCA Ca^2+^-ATPase. Note that while some heat is liberated by these ATPases, most muscle thermogenesis is via mitochondrial catabolic processes which generate the proton motive force required for ATP synthesis. **(C)** Mammalian phylogenetic tree denoting potentially relevant SLN residue substitutions (black), start codon mutations (blue), and deletions (red) based on data in Gaudry et al. (2017). SLN residue replacement and deletion events are not dated and are placed on the phylogenetic branches in sequential order. The height of collapsed branches (gray triangles) is proportional to the number of SLN sequences available for each clade. Note that published functional studies pertaining to SLN-NST have been restricted to a few model species in the Glires clade (predominantly mice). Silhouettes are taken from phylopic.org (image credits: megabat, Oren Peles/vectorized by Yan Wong; rabbit and sloth, Sarah Werning; https://creativecommons.org/licenses/by/3.0/).

Precise control of cytosolic Ca^2+^ levels underlies the modulation of optimal contraction strengths, frequencies, and relaxation rates by cardiac and skeletal muscle over a wide range of activities. This regulation is largely achieved by metering the release of SR Ca^2+^ stores into the cytosol via ryanodine receptor channels and the subsequent uptake of Ca^2+^ by sarco/endoplasmic reticulum Ca^2+^-ATPase (SERCA) pumps. Briefly, SERCA harnesses the phosphate bond energy of one ATP molecule to translocate two Ca^2+^ ions into the SR lumen, thereby generating a ~15,000-fold (1.5 mM vs. 0.1 μM) lumen-to-cytosol Ca^2+^ concentration gradient in resting muscle (Toyoshima and Inesi, 2004). Several single-pass transmembrane peptides—including phospholamban, myoregulin, and sarcolipin (SLN)—interact with SERCA isoforms to modify Ca^2+^ uptake in a tissue specific manner. SLN, for example, is a relatively short (31 amino acid) helical peptide that was initially shown to alter Ca^2+^ uptake kinetics by inhibiting SERCA1a and SERCA2a activity in the atria and skeletal muscles of mammals (Odermatt et al., 1998; MacLennan et al., 2003). Subsequent *in vitro* evidence that SLN increases the heat generated by SERCA by partially uncoupling Ca^2+^ re-sequestration from ATP hydrolysis in rabbit and mouse skeletal muscle (Smith et al., 2002; Mall et al., 2006) has led to the hypothesis that SLN contributes to non-shivering thermogenesis (NST) *in vivo* (Bal et al., 2012). It should be noted that this proposed heat generating process is mechanistically distinct from, and hence unrelated to, known Ca^2+^-linked thermogenic processes in skeletal muscle, which will not be further discussed here, such as futile Ca^2+^ cycling in the non-contractile extraocular muscles of regionally endothermic billfishes and ryanodine receptor mutations associated with malignant hyperthermia. While there remains a lack of consensus regarding adaptive muscle NST in mammals by any mechanism, the proposal that SLN increases energy turnover in the skeletal muscles where it is expressed has generated substantial interest in the biomedical research community as a potential target for obesity and other metabolic syndromes. To date, however, studies on the structure, function, and regulation of SLN, its interaction with SERCA, and its potential role in facultative muscle NST predominantly remain limited to a few model systems that reside within the same mammalian clade (Glires; rodents and rabbits). While this work is tantalizing, several key mechanistic aspects remain unanswered such as (1) whether uncoupling of Ca^2+^ transport from ATP hydrolysis in SERCA primarily arises from slippage of the SERCA-bound Ca^2+^ ions into the cytosol after ATP hydrolysis or from passive leak of luminal Ca^2+^ back into the cytosol through SERCA (Figure 1A), (2) what region(s) and/or residue(s) of SLN and SERCA are involved in this uncoupling, and (3) whether SLN-mediated Ca^2+^ uncoupling occurs in resting muscle when SR luminal Ca^2+^ concentration is high and cytosolic [Ca^2+^] is low, or in actively-contracting and/or shivering muscle when cytosolic Ca^2+^ is elevated (de Meis, 2001; Smith et al., 2002; Inesi and Tadini-Buoninsegni, 2014). Also unclear are broader questions pertaining to how SLN-mediated Ca^2+^ uncoupling evolved, and its occurrence and potential thermogenic importance—i.e., how much heat is produced relative to shivering—across the mammalian phylogeny (and beyond).

## Heat yes, but how much?

It is imperative to delineate the potential amount of heat generated via SLN-mediated NST from that of other ATP consuming processes coupled to Ca^2+^ turnover during a muscle contraction-relaxation cycle. These include activation of sarcolemma Na^+^-K^+^-ATPases for membrane repolarization, myosin-ATPases for cross-bridge relaxation, and SERCA Ca^2+^-ATPases for Ca^2+^ sequestration (Figure 1B). As SLN is not associated with the former two ATPases, back of the napkin calculations suggest its thermogenic contribution is limited to a small fraction of that liberated by shivering. For simplicity, if we arbitrarily assume that 1,000 Ca^2+^ ions are released into the cytosol following nervous activation, then transporting these Ca^2+^ ions back into the SR will hydrolyze 500 ATP (assuming no Ca^2+^ backflux or leak), with an additional 750–1,167 ATP hydrolyzed by the Na^+^-K^+^ and myosin ATPases (these two ATPases are estimated to contribute ~60–70% of the energetic cost of an isometric muscle contraction; Barclay et al., 2007). For SLN to not interfere with muscle relaxation speeds, and hence maximal contraction frequencies—an especially important consideration for small mammals—SLN uncoupling is likely to predominantly operate when SR luminal concentrations are elevated (Inesi and Tadini-Buoninsegni, 2014). Thus, assuming Ca^2+^ slippage/leak occurs at a cytosolic [Ca^2+^] low enough to preclude cross-bridge formation—say below 100 Ca^2+^ ions in our example—and that the Ca^2+^/ATP coupling ratio is lowered by 35% (Bombardier et al., 2013), then this mechanism would only hydrolyze an additional ~25 ATP, or ~2% of the heat produced by a single contraction-relaxation cycle. Results of *in vitro* biochemical assays demonstrate that SERCA activity is almost undetectable at Ca^2+^ levels found in resting muscle (Gorski et al., 2013; Sahoo et al., 2013). Nonetheless, even if we assume that SLN lowers net Ca^2+^ pumping efficiency to zero in this state, then >3-fold more Ca^2+^ ions than are released during a single muscle contraction would need to “slip” for SLN to generate an equivalent amount of heat within the same timeframe. Of course, this latter scenario also requires that cytosolic Ca^2+^ levels remain below the contraction threshold, whereby few SERCA Ca^2+^ binding sites would be populated relative to that following muscle contraction (Barclay et al., 2007). Taken together, these calculations question whether SLN-NST can contribute meaningfully to thermogenesis relative to shivering and other non-shivering processes (e.g., brown adipose tissue).

## Evidence for SLN-mediated non-shivering thermogenesis is inconclusive

Crucially, heat released via the SLN and SERCA complex has been shown to increase linearly with SLN concentration *in vitro* (Mall et al., 2006), which current data suggests varies markedly among skeletal muscles in rodents. Briefly, moderate concentrations of SLN are present in small muscles of mice such as the soleus and diaphragm that are predominantly composed of slow-twitch oxidative fibers, though (nearly) undetectable in fast-twitch glycolytic fibers that form the bulk of the limb musculature (e.g., quadriceps; Vangheluwe et al., 2005; Babu et al., 2007; Bombardier et al., 2013). Consequently, any NST linked to SLN may be expected to be imperceptible in these small mammals. This conclusion is in line with recent work on SLN knock-out mice that revealed no differences in body mass, food intake, or resting whole body and isolated soleus muscle O_2_ consumption rates relative to wild-type littermates, despite indications that SLN reduced the apparent Ca^2+^/ATP coupling ratio within the soleus (but not fast-twitch extensor digitorum longus muscles) by up to 35% (Bombardier et al., 2013). Similarly, SLN overexpressing mice exhibited no differences in body mass or energy expenditure, even on high fat diets, relative to wild-type individuals whose muscles contained ~25-fold lower SLN concentrations (Butler et al., 2015). It should be noted, however, that these findings are not universal, as pair-fed SLN knock-out mice gained weight relative to wild-type mice, while SLN overexpression resulted in weight loss and an increased rate of O_2_ consumption (Maurya et al., 2015). The basis for these contradictory findings remains unclear, though differences in O_2_ consumption may have arisen in part due to values being presented in a mass-specific basis for the latter study. Nonetheless, results of the Maurya study are ostensibly bolstered by several mouse studies that contend that loss of SLN hinders muscle-based NST (Bal et al., 2012, 2017; Rowland et al., 2015), although it is also conceivable that the observed effects of SLN ablation arise from an impaired shivering response, altered Ca^2+^ signaling pathways, and/or compromised Ca^2+^ handling.

Unfortunately, *in vivo* experiments in support of muscle NST that involved curare (e.g., Bal et al., 2012) are inconclusive as none of the animals required artificial respiration, thus indicating that shivering may not have been completely blocked. Additional support for a thermogenic role for SLN is that cold acclimation mitigates the natural developmental reduction in SLN levels in neonatal mice and results in elevated SLN transcription in slow-twitch adult skeletal muscle (Pant et al., 2015). However, this interpretation is complicated by studies conducted on non-cold stressed wheel running mice whose soleus SLN mRNA expression profiles show a distinct exercise effect, being 2- to 3-fold higher than found in sedentary mouse muscle (de Snoo, 2009). Consequently, the observed elevations in muscle SLN levels in cold challenged mice may simply reflect a shivering induced “training effect.” While additional research in this area is required, the findings of the wheel running mice studies are not consistent with an adaptive thermogenic role for SLN, as elevated ATP hydrolysis due to futile Ca^2+^ pumping would be expected to impair exercise performance (see below).

## SLN expression and evolutionary analyses

Previous work has shown SLN mRNA and/or protein levels to be several magnitudes higher in both slow and fast twitch skeletal muscles of rabbits, pigs, dogs, and humans than lab rodents, leading to suggestions that SLN-NST may be of greater thermogenic importance in larger mammals (Barbot et al., 2016). However, given that larger mammals also possess lower surface-area-to-volume ratios, thicker insulation, and the ability to further minimize heat loss via countercurrent heat exchangers, this contention is puzzling. Similarly, implications that larger mammals may exhibit a stronger reliance on SLN-NST due to small (or even absent) depots of thermogenic brown adipose tissue (BAT) are unsatisfactory, as it is precisely for the reasons outlined above that these species have a reduced reliance on BAT thermogenesis (i.e., a reduction in BAT mass does not need to be counterbalanced by other heat generating mechanisms). Indeed, recent work has illustrated that ancient magnitude increases in body mass in five mammalian lineages [cetaceans (whales and dolphins), elephantids, sirenians (sea cows), hyracoids (hyraxes), and equids (horses and kin)] were temporally coupled with inactivation of thermogenic *UCP1* (Gaudry et al., 2017), suggesting that costs of maintaining an elevated thermogenic capacity outweighed its benefits.

If SLN plays a prominent thermoregulatory role in mammals, then this locus may be expected to have evolved under relaxed selection or potentially even be pseudogenized in large or tropically distributed species and/or mammalian lineages lacking a functional UCP1 protein. The latter assemblage offers particularly good model systems as members of some UCP1 lacking lineages (cetaceans, woolly mammoths, Steller's sea cows, and horses) have nonetheless evolved extreme cold tolerance while others (pangolins, xenarthrans, hyraxes, pigs, and extant sea cows) are notoriously cold intolerant (even often as adults). In contrast to such expectations, not only is the SLN locus functionally intact in each of these cases (i.e., able to encode a ~31 unit peptide), but its primary structure has remained exceptionally well conserved despite highly divergent body sizes and thermoregulatory capacities among species. For example, UCP1-lacking elephants, whales, pigs, and hyraxes all possess *identical* SLN peptides to those of rabbits, while SLN sequences of “proto-endothermic” tenrecs and golden moles precisely match those of cold tolerant deer mice and meadow voles (*cf*. Figure S4 of Gaudry et al., 2017). This remarkable degree of sequence conservation—over evolutionary timescales exceeding 65 million years, and in large bodied and tropical species for which NST should have no apparent adaptive benefit—is not readily compatible with a thermogenic function. Intriguingly, the start codon of SLN is mutated in weakly endothermic sloths (Figure 1C), which moreover have nonfunctional UCP1 (Gaudry et al., 2017), though its primary structure remains highly conserved; whether the start codon mutation inhibits protein translation or is rescued by alternative splicing remains unknown and should be examined.

While there is evidence that SLN interaction with SERCA results in an elevated rate of ATP hydrolysis *in vitro*, taken together, the above findings argue that the primary role of SLN in skeletal muscle of mammals is not thermoregulatory in nature. Nonetheless, SLN expression and *in vitro* studies on a range of large-bodied mammals, including species lacking functional UCP1, are required to confirm or refute the above contentions. An examination of representative marsupials and monotremes are also required to assess the thermogenic potential of SLN across Mammalia and better pinpoint if and when the proposed thermogenic function arose in evolutionary time. Finally, it is worth noting that the high expression level of SLN in mammalian atria—the concentration of which is 10-to 1,000-fold higher than in the soleus of rodents (Babu et al., 2007; Bombardier et al., 2013)—is also difficult to envision within a thermogenic framework, though in line with a role in modulating intracellular Ca^2+^ homeostasis by modulating the (apparent) Ca^2+^ affinity of SERCA. Indeed, both elevated and lowered atrial SLN concentrations are linked to cardiac pathologies (Babu et al., 2007).

## How is SLN regulated?

Surprisingly, the mechanism underling SERCA inhibition by SLN is also equivocal. For example, some labs report a decrease in SERCA's apparent Ca^2+^ affinity in the presence of SLN (Hellstern et al., 2001; Buck et al., 2003; Buffy et al., 2006; Gorski et al., 2013) whereas others do not observe a change in Ca^2+^ affinity and only observe a decrease in Ca^2+^ uptake (Smith et al., 2002; Sahoo et al., 2013). There are several potential reasons for these discordant observations including the use of different assays to measure ATP hydrolysis as well as the use of different test species employed (with different SLN and SERCA sequences). It is essential to both understand and reconcile these inconsistencies to determine the mechanism of inhibition, and whether or not this process is linked to thermogenic Ca^2+^ uncoupling/leak. Similarly, comparative (and, potentially, site-directed mutagenesis) studies are also required to elucidate what component of the SLN peptide is involved in thermogenesis. Two recent studies have implicated the cytosol-oriented N-terminus to underlie Ca^2+^ uncoupling (Sahoo et al., 2015; Autry et al., 2016). Specifically, rabbit SLN residues Glu^2^ and Glu^7^ are predicted to form salt-bridges with Arg^324^ and Lys^328^ of rabbit SERCA1a, thereby perturbing one of the SERCA Ca^2+^ binding sites (E^309^ on helix M4), although only Glu^7^ was predicted as necessary to induce the uncoupling structural change (Autry et al., 2016). Dicke (2017) has questioned the role of this region in uncoupling due to a substantial degree of sequence variability among mammals (e.g., MGINTRE^7^ in catarrhine primates vs. MERSTQE^7^ in most rodents; Gaudry et al., 2017), and it remains unknown whether *in vitro* SLN Ca^2+^/ATP uncoupling is compromised by the Glu^2^ → Gly^2^ replacement in human, carnivore, and aardvark SLN, the Glu^2^ → Asp^2^ replacement in vesper bats, or the Glu^7^ → Asp^7^ replacement in sloths, dugonid sea cows, and false vampire bats (Figure 1C). It should be further noted that *Xenopus* and zebrafish—for which a non-shivering thermogenic mechanism would be most unexpected—possess the identical SLN N-terminal MERSTQE motif found in rodents (their SERCA proteins also contain Arg^324^ and Lys^328^). By contrast, the luminal C-terminal sequence (^27^RSYQY^31^)—which underlies both the proper SR/ER cellular localization of SLN and its apparent inhibition of SERCA (Gramolini et al., 2004; Gorski et al., 2013)—is nearly 100% conserved among mammals, and thus may be a better candidate for an uncoupling function (Dicke, 2017). Importantly, two C-terminal residues (Arg^27^ and Tyr^31^) that are proposed to be essential for SLN inhibition (Gorski et al., 2013) are only found in mammals, with other vertebrates having Lys^27^ and Glu/Asp^31^ at these sites, except falcons which have Gln^31^ (Montigny et al., 2014; Gaudry et al., 2017). Interestingly, Tyr^31^ → Ser^31^ substitutions appear to have evolved independently at least three times in mammals (twice in bats and again in flying lemurs), while this C-terminal residue is deleted in equids (Figure 1C). These species accordingly provide natural model systems to help evaluate the potential role of the cytosolic (N-terminus) and luminal (C-terminus) regions of SLN in both decreasing SERCA's apparent Ca^2+^ affinity and uncoupling of Ca^2+^ transport.

In addition to supplying sufficient heat to help maintain thermal balance in sub-thermoneutral temperatures, a key feature for any adaptive, facultative thermogenic process is the ability to rapidly activate it once the need arises and inactivate it once additional thermogenesis is no longer required. If, for example, SLN-NST is simply an unregulated by-product associated with Ca^2+^ pumping during muscle contraction, then this mechanism would become maladaptive in many circumstances such as during exercise in temperatures near or above the lower limit of the thermoneutral zone, as this supplemental heat production would tax heat-dissipating processes thereby promoting hyperthermia-induced muscle fatigue and impaired motor performance (Silva, 2011; Nybo et al., 2014). To our knowledge, no studies have yet demonstrated that SLN-NST can be activated/inactivated independently from muscle contraction, let alone have identified the molecular mechanism by which this process can be mediated. While phosphorylation of one or more residues (Ser^4^ and/or Thr^5^) within the cytosolic domain have been shown to relieve the inhibitory effect of SLN on cardiac SERCA pumps (Gramolini et al., 2004; Bhupathy et al., 2009), likely via structural changes to the SLN-SERCA complex, it remains unknown whether this mechanism alters uncoupling activity (Autry et al., 2016; Barbot et al., 2016). However, primary sequence analysis of available data reveal that either one or both of Ser^4^ and Thr^5^ are—with one exception (equids)—universally present from fish to mammals (Barbot et al., 2016; Gaudry et al., 2017), indicating that this inhibitory mechanism of control is likely both ancient and highly conserved. Key insights into this regulation may thus be provided by studies on horses/donkeys whose SLN peptides (in addition to lacking the C-terminal Tyr^31^ residue; see above) also lack the potential for reversible phosphorylation (one of the two residues at positions 4–5 is deleted, while the other exhibits a dephospho-mimetic Thr→Arg substitution; Figure 1C).

In addition to phosphorylation as a post-translational modification, it was recently shown that Cys^9^ of native SLN from rabbits and pigs was S-palmitoylated and S-oleylated (Montigny et al., 2014), and that depalmitoylation treatment increased Ca^2+^-ATPase activity of rabbit SR by 30%. Rodents, by contrast, possess Phe^9^ and show a smaller increase in Ca^2+^-ATPase activity following depalmitoylation (20%; Montigny et al., 2014), suggesting a potential (albeit limited) role for this residue position in the regulation of SLN activity. However, the presence of Cys^9^—found in mammals ranging from rabbits, bats, kangaroos, and pangolins to elephants and whales (Figure 1C)—does not appear to be linked with either body mass or metabolic intensity. While this phylogenetic distribution is consistent with a pattern of both repeated (convergent) evolution of Cys^9^ and its reversion back to the ancestral Phe^9^ found in the majority of vertebrate species, the potential physiological and thermogenic effects of this residue replacement are yet to be assessed.

## Conclusions

While there can be little doubt that the interaction of SLN with SERCA lowers the metabolic efficiency of Ca^2+^ transport *in vitro*, unequivocal support for an adaptive thermogenic role *in vivo* is lacking. Suggestions that SLN plays any meaningful thermogenic role in larger bodied mammals (including humans) or non-mammalian vertebrates (e.g., birds) is also without empirical evidence and remains purely speculative. Comparative studies taking advantage of naturally occurring variability in SLN concentrations and primary sequence among vertebrates provide ideal avenues to explore the function of this regulatory peptide, and its evolution.

## Author contributions

KC drafted the manuscript, prepared the figures, and approved the final version. AD contributed to manuscript writing, provided important interpretations, critically revised the work, and provided final approval of the opinion content.

### Conflict of interest statement

AD was employed by Fish & Richardson P.C. at the time of writing. The ideas and opinions in the article are her own, and predominantly arose from biophysical research on SLN interactions with SERCA at the University of Minnesota during the course of her Ph.D. These viewpoints have not been vetted with the law firm where she works or its clients, and do not represent the positions of the firm, its lawyers, or any of its clients. None of this writing is intended as legal advice and it should not be taken as such. The remaining author declares that the research was conducted in the absence of any commercial or financial relationships that could be construed as a potential conflict of interest.
